# Chemical and Sensory Properties of Waffles Supplemented with Almond Skins

**DOI:** 10.3390/molecules28155674

**Published:** 2023-07-27

**Authors:** Ivo Oliveira, Beatriz Marinho, Urszula Szymanowska, Monika Karas, Alice Vilela

**Affiliations:** 1 CITAB, Centre for the Research and Technology of Agro-Environmental and Biological Sciences and Inov4Agro, Department of Applied Biology, Institute for Innovation, Capacity Building and Sustainability of Agrifood Production, School of Life and Environmental Sciences, University of Trás-os-Montes and Alto Douro, Quinta dos Prados, 5000-801 Vila Real, Portugal; ivo.vaz.oliveira@utad.pt; 2 University of Trás-os-Montes and Alto Douro, Quinta dos Prados, 5000-801 Vila Real, Portugal; beatrizfariamarinho@gmail.com; 3 Department of Biochemistry and Food Chemistry, University of Life Sciences, Skromna 8, 20-704 Lublin, Poland; urszula.szymanowska@up.lublin.pl (U.S.); monika.karas@up.lublin.pl (M.K.); 4 Chemistry Research Centre (CQ-VR), Department of Agronomy (DAgro), School of Agrarian and Veterinary Sciences (ECAV), University of Trás-os-Montes and Alto Douro, Quinta dos Prados, 5000-801 Vila Real, Portugal

**Keywords:** waffles, almond skin, phenolic compounds, antioxidant activity, soluble sugars, starch

## Abstract

Almonds are one of the most produced nuts worldwide and numerous studies have shown that they have nutritional and medicinal characteristics, which gives them the possibility of being applied in various products. However, several by-products are generated during their production, which have characteristics of interest but remain underutilised, namely, the almond skins. This work aimed to study samples of waffles supplemented with almond skins. The waffles were evaluated for their total polyphenol content, antioxidant capacity, total flavonoids, *ortho*-diphenols, soluble sugars, starch, texture, and colour. They were also sensorially evaluated using a panel of tasters specialised in this type of evaluation and a quantitative descriptive analysis test (QDA) sensory test. The results showed that the waffles with the highest levels of phenolic compounds as well as the highest antioxidant activity (by the ABTS, DPPH, and FRAP methods) were the waffles supplemented with 10% almond skin. The total phenol contents obtained for the prepared extracts varied between 0.127 mg GAE/g and 0.415 mg GAE/g, the flavonoid contents ranged from 0.067 mg CAE/g to 0.339 mg CAE/g and the *ortho*-diphenol contents varied between 0.163 mg ACE /g and 0.303 mg ACE/g. Regarding the quantification of soluble sugars, the values were presented in percentage of fresh weight, and ranged from 30.148 to 38.054%; regarding the quantification of starch, the percentages varied from 14.488 to 21.982%. Sensorially, we verified that the samples were statistically different in terms of the descriptors “colour”, “roasted aroma”, and “dissolubility”, with a higher score in these descriptors for the waffles with 10% of almond skin. This process of obtaining waffles, which can be industrialised, is interesting from both a nutritional point of view and for the possibility of creating new, differentiated, and innovative products.

## 1. Introduction

The almond tree (*Prunus dulcis*), belonging to the Rosaceae family like the apple tree, pear tree, and plum tree, is one of the most popular dry fruit trees, occupying first place in terms of production worldwide [1]. 

**Figure 1:**
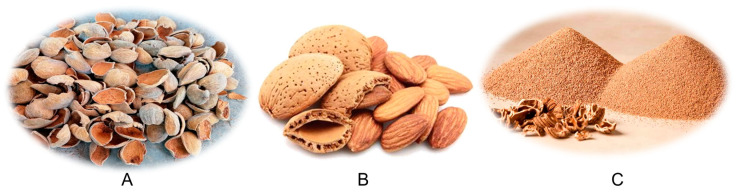
Almond by-products. Hulls (**A**), shells (**B**), skins, and skin powder (**C**).

Currently, almond production (over 3,993,998 t, in 2021) generates large amounts of by-products (Figure 1). Although these have still been little explored, they are made up of the outer husk, inner husk, and pellicle (skins), with different physicochemical properties, where the heaviest material is represented by the external husk, which in terms of the total fresh weight represents 52%, while the inner rind and kernel (including the skin) represent 33% and 15%, respectively [2].

As a result, due to the quantities produced, the almond processing industry saw the need to consider new ways of using/treating by-products. Currently, they are mostly used in livestock feed, obtaining energy in gasification plants as well as in the production of activated charcoal from the husks [3,4].

The almond skin, one of the by-products obtained during processing, presents compounds that can be used in the areas of medicine, pharmaceuticals, and food. This contains approximately 50–75% of the total phenols contained in the almond fruit [5], which demonstrates its enormous applicability in the extraction of phenolic compounds/antioxidants, antimicrobials, and antiviral activity, among others [6,7].

In the last years, several works regarding almond skin have shown that polyphenols are present in large amounts including flavonoids, flavanones, simple phenolic acids, and flavonols, compounds known to decrease the risk of developing chronic inflammation and modulate the immune system response [8,9]. Almond skin is rich in fibre (52.6 g/100 g) and lipids (21.3 g/100 g), mainly of mono and polyunsaturated fatty acids with a low content of protein (11 g/100 g) and carbohydrates [4]. Currently, the almond industry uses blanching with hot water and steam due to their ability to improve product quality, increase yield, and facilitate the processing of products with different thermal properties [10].

Blanching results in a loss of substances, resulting in a higher content of compounds in the bleaching water compared to what is expected to be found in the skin [11]. However, this significant loss of compounds to the bleaching water does not hinder the possible applications of the almond skin, given that blanched skins still present positive results in different research areas [12].

Over the last few years, consumers’ attitudes towards food have undergone significant changes, with a greater interest in the demand for and consumption of healthier and more natural foods. This situation results from consumers becoming aware that by improving their eating habits, they are contributing to a better functioning of the organism, and therefore to their health status [13,14], pushing the industry to develop new food products to respond to the increased demand for this type of food. Thus, several foods entitled “functional foods” were and are being developed. These foods, in addition to providing the expected nutrients, contain compounds that contribute to improving health and reducing the risk of disease, having additional benefits that originate from the presence of some components such as bioactive compounds, probiotics, unsaturated fatty acids, and dietary fibre [15].

In general, different parts of the fruit have been used such as the peel, pulp, and core in the form of flour. Several studies in the literature have described the use of this type of flour (using fruit by-products), for example, prepared from banana peels [16], Congo peas [17], pomegranate peels [18], and almond [19] in the preparation of biscuits.

It should be noted that according to Dhillon et al. [19], the consumption of almond-supplemented waffles, over a period of 8 weeks, boosted the increase in aconitic, citric, isocitric, and succinic acids, which suggests that almond consumption stimulates the Krebs cycle.

Waffles are a convenient and very popular product, rich in energy but lacking the phytonutrients related to health benefits, providing a great research line, upcycling residues, and, at the same time, enhancing the properties and characteristics of a well-accepted product. Therefore, the general objective of this work was to evaluate the potential of incorporating almond skin in waffles through the study of the sensory and chemical properties (bioactive content and antioxidant activity) of waffles supplemented with different proportions of almond skin.

## 2. Results and Discussion

### 2.1. Analysis of Bioactive Compound Content and Antioxidant Activity

#### 2.1.1. Total Phenol, Flavonoids, and *Ortho*-Diphenols Content Analysis

**Figure 2:**
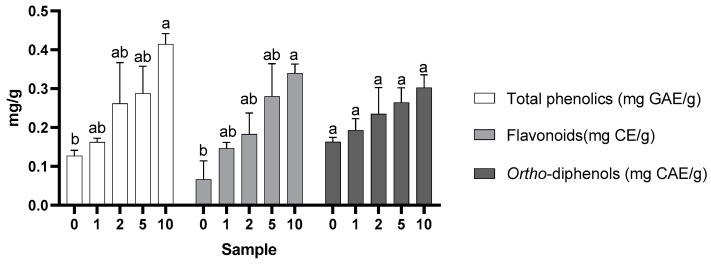
Total phenol, flavonoid, and *ortho*-diphenol content in the waffles (mean + SD). Different letters indicate statistically significant differences between samples (*p* ≤ 0.05, Tukey’s tests).

The total phenol, flavonoid, and *ortho*-diphenol contents in waffles are shown in Figure 2. For the total phenols, the results ranged between 0.127 (control sample—0%) and 0.415 mg (sample with 10% almond skins) GAE/g sample. Between the samples of 1%, 2%, and 5%, there were no significant differences in the total phenol content, contrary to the control sample and the 10% supplemented sample.

For the flavonoid content (Figure 2), the results alternated between 0.067 (control sample) and 0.339 (sample with 10% almond skins) mg CE equivalent/g sample. The values showed the same behaviour as that already mentioned for the total phenol content.

The results of the *ortho*-diphenols (Figure 2) ranged from 0.163 (control sample) to 0.303 (10% almond skins) mg CAE equivalent/g sample. Although these values differed numerically, statistically there were no significant variations in the five samples, and there was no apparent influence of the use of added almond skins, although there was a noticeable tendency for an increase in the content of the samples with higher percentages of supplementation.

The increase in the phenolic content with the addition of almond skin is probably related to the high content of these compounds present in the skin [5]. Similar results showing an increase in the phenolic compound content with the incorporation of alternative ingredients in waffles have also been described by other authors. The use of raspberry juice or pulp, according to Szymanowska et al. [20,21], increases the total phenols with increasing juice or pulp added to the waffles. Similarly, Urganci and Fatma [22] studied the influence of increasing pomegranate peel flour in cookie preparation and observed that the phenol content increased as the amount of peel flour incorporated into the waffles increased. The influence of the addition of almond skin on the content of flavonoids and *ortho*-diphenols is also likely linked to the higher presence of these compounds in the almond’s skin [2].

Salem et al. [23] prepared waffles with a mixture of tomato pulp and pomegranate seed flour, supplemented with different percentages of this mixture (control, 2.5%, 5%, and 10%). A higher flavonoid content was recorded in the samples where the flour mixture was added at a higher percentage compared to the control sample. Similarly, the work by Szymanowska et al. [20,21] showed that the higher the proportion of raspberry juice or pulp incorporated into the waffles, the higher the flavonoid content.

Regarding the content of *ortho*-diphenols, although the values differed numerically, statistically there were no significant variations in the five samples, and there was no apparent influence of the use of added almond skin, although there was a noticeable tendency for an increase in content in the samples with higher percentages of supplementation.

#### 2.1.2. Analysis of Antioxidant Capacity by the DPPH, ABTS, and FRAP Methods

**Figure 3:**
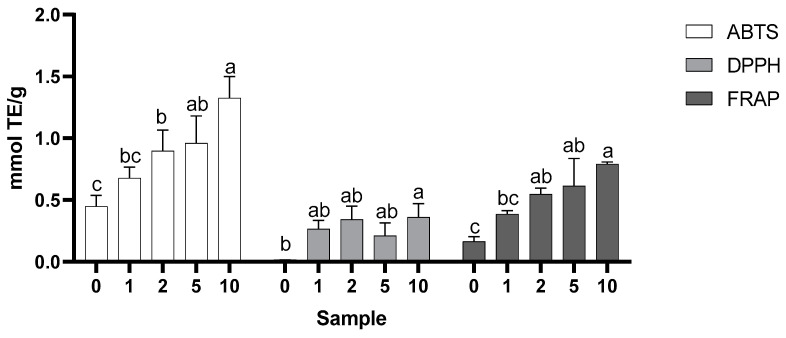
Antioxidant activity was measured using the ABTS, DPPH, and FRAP methods of the waffles (mean + SD). Different letters indicate statistically significant differences between samples (*p* ≤ 0.05, Tukey’s tests).

The antioxidant capacity determined by the DPPH, ABTS, and FRAP methods in waffles is shown in Figure 3.

For the antioxidant capacity determined by the DPPH method (Figure 3), the results showed an activity ranging between 0.015 and 0.360 mmol ET/g of the sample. In the graphical representation, it was possible to observe an increase in the antioxidant capacity of the samples containing a higher percentage of the added almond skins. The results showed that the cookie supplemented with 10% almond skin displayed an ability to neutralize the DPPH˙ radical that was effectively higher than the control one.

For the ABTS method (Figure 3), the results showed an activity ranging from 0.449 to 1.33 mmol ET/g of sample. In general, higher values were recorded in the waffles with a higher percentage of skins.

The results of the FRAP method (Figure 3) alternated between 0.164 and 0.791 mmol ET/g of the sample. In the graphical representation, it was possible to observe an increase in antioxidant capacity in the waffles containing a higher percentage of the added almond skins. The 10% sample showed significantly higher results than the control and 1% samples, while there were no significant variations in the others.

Szymanowska et al. [20,21] prepared waffles supplemented with raspberry pulp in different amounts (control, 10%, 20%, 30%, 50%, and 75%) and found that the ability of the ethanolic extracts to neutralize the DPPH˙ radical increased as the concentration of pulp added to the waffles increased. In our work, the antioxidant activity measured by the DPPH method was positively correlated with the total phenol content (R = 0.549; *p* = 0.033) and flavonoid content (R = 0.612; *p* = 0.015). These correlations between the bioactive content and antioxidant activity evaluation using the DPPH method have previously been found in almond samples [24,25] and in waffles supplemented with raspberry juice and pulp [20,21].

Ogunjobi and Ogunwolu [26], when studying the influence of increasing the amount of apple powder flour in crackers, concluded that the antioxidant activity, measured using the ABTS methodology, increased when increasing the amount of flour. Similarly, the work of Szymanowska et al. [20,21], using a similar cookie formulation but with the addition of raspberry juice or pulp, also recorded an increase in the antioxidant activity measured by this method with an increasing proportion of raspberry added. The antioxidant activity measured by the ABTS method was positively correlated with the content of total phenols (R = 0.658, *p* = 0.008). Similarly, the content in flavonoids (R = 0.697, *p* = 0.004) and in *ortho*-diphenols (R = 0.726, *p* = 0.002) were also correlated by the activity measured. These correlations of ABTS, recorded activity, and bioactive content have also been previously found in almond samples [24,25] and in waffles supplemented with raspberry juice and pulp [20,21].

Analogously, Mahloko et al. [16], when preparing waffles with a mixture of banana and prickly pear (*Opuntia ficus-indica*) flours, supplemented with different percentages of this mixture (control, 4%, 8%, and 16%), found that the FRAP values ranged between 0.59 and 0.71 mmol TE/g sample, in which the 16% sample recorded the highest value, in contrast to the control, which recorded the lowest. These results agree with those obtained in this work. In the present work, the antioxidant activity measured by the FRAP method was positively correlated with the total phenol content (R = 0.791; *p* = 0.001), the flavonoid content (R = 0.688; *p* = 0.005), and *ortho*-diphenols (R = 0.523; *p* = 0.046).

### 2.2. Quantification of Starch and Soluble Sugars

**Figure 4:**
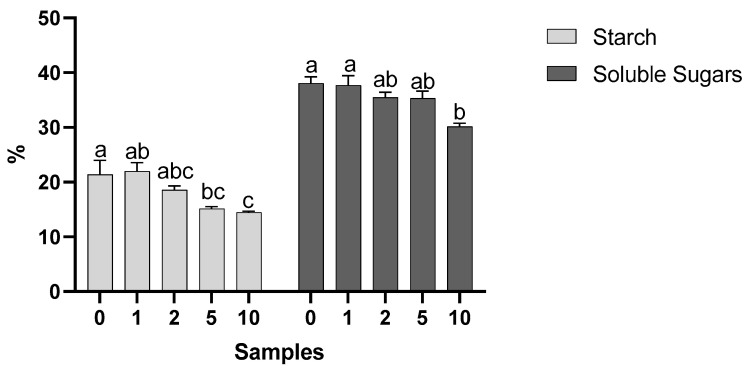
Percentage of starch and soluble sugars recorded in the waffles (mean + SD). Different letters indicate statistically different differences (*p* ≤ 0.05, Tukey’s tests).

The waffles recorded starch values (percentage of fresh weight) between 14.488 and 21.982% (Figure 4). The 10% sample showed significantly lower results than the control sample. This was due to the formulation of the waffles, where the blended flour was replaced by almond skins at 0% (control), 1%, 2%, 5%, and 10%, which means that the control sample had a higher amount of flour, and consequently, starch. The values of soluble sugars were converted into fresh weight percentages and comprised values from 30.148 to 38.054%. Although the results obtained were not significantly different among the samples, except for the control and 10% sample, the sample with the highest amount of soluble sugars was the control.

Although the results obtained were not significantly different between the samples, contrary to some parameters previously studied, the sample with the highest amount of soluble sugars was the control. Wheat flour has soluble sugars in its composition [27], and considering that with the increase in the almond skin content, the amount of flour used is reduced, it may justify the fact that the percentage of sugars also decreases. The 10% sample showed significantly lower results than the control sample. This was due to the formulation of the waffles, where the blended flour was replaced by the almond skins at 0% (control), 1%, 2%, 5%, and 10%, meaning that the control sample had a higher amount of flour, and consequently, starch. Identically, Ng et al. [28] baked waffles where they replaced the flour with mushroom-oyster powder (*Pleurotus ostreatus*) at 0% (control), 4%, 8%, and 12%. The starch content in the control sample was the highest (39.21%), while the content for sample 12% (33.94%) was the lowest. These results support those obtained in this work.

### 2.3. Texture Evaluation

**Figure 5:**
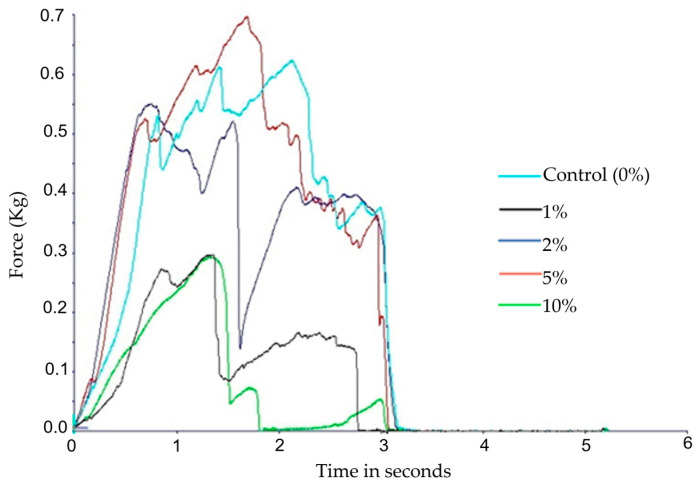
Texturogram of the waffles with different concentrations of almond skins.

A texturometer with a 5 kg load cell was used for texture evaluation, and penetration tests were performed using a 2-mm diameter cylindrical stainless-steel probe. The results for the different samples can be seen in this texturogram graph, where the maximum force required (peak of the graph) to break the waffles can be observed (Figure 5).

By analysing Figure 5, it is possible to see that the force required for the waffle to break did not seem to be correlated with the concentration of almond skins in its formulation. It might be expected that a waffle with more skins would be crumblier and easier to break, a textural change already recorded with the use of almond skin in biscuits [4]. However, one explanation for this phenomenon may be the fact that the waffles varied greatly in weight once they were made manually, without the use of specific normalised and standardised instruments. The weight also did not correlate with the concentration of almond skins (Table 1). Studies by Sudha et al. [29] and Ajila et al. [30] obtained results similar to ours.

**Figure 6:**
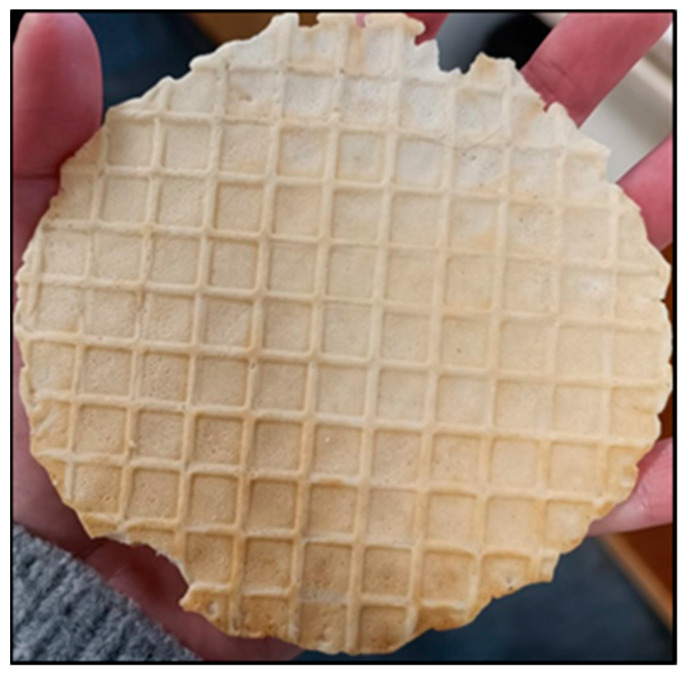
Visual evaluation of the outside of the control waffle.

Moreover, there may be differences in the thickness of the waffles that influenced the evaluation of this parameter. The differences in weight and thickness were because they were prepared in the laboratory, and not in an industrial environment (as mentioned before) with standardised equipment. Additionally, the waffles did not have a uniform surface as they had ridges (Figure 6), and in these ridges, the force required to break the waffle will probably be greater.

### 2.4. Instrumental Colour Evaluation

**Figure 7:**
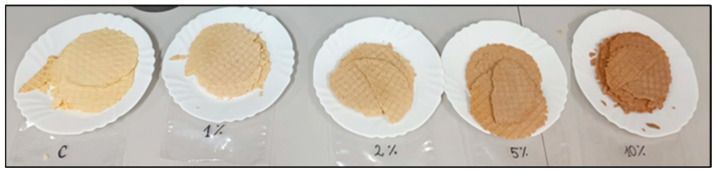
The visual appearance of the evaluated waffles.

The colour was evaluated in a three-dimensional coordinate system using the L*, a*, b* model [24]. In Figure 7, is possible to see, with the naked eye, the differences between the waffle samples and how the colour intensity increased from the control (C) to the 10% almond skin sample.

**Figure 8:**
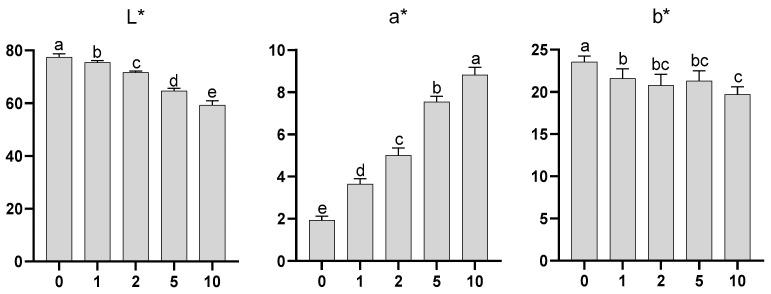
Colour parameter values (L*, a*, and b*)—mean + SD—for the evaluated waffle samples. Different letters indicate statistically significant differences between samples (*p* ≤ 0.05, Tukey’s tests).

According to the three-dimensional coordinate system, the values for the luminosity parameter (L*) range from 0 (black) to 100 (white), for the a* coordinate from green (when negative) to red (when positive), and for the b* coordinate from blue (when negative) to yellow (when positive). In our work, the results obtained for the parameters L*, a*, and b* are shown in Figure 8.

For the parameter a*, the values were significantly different among all samples (Figure 8), and there was an increase in this coordinate with the increase in the proportion of almond skin. Note that the values were low, close to 0, indicating that there were no pronounced green or red shades in the waffles. Regarding the b* coordinate, significant differences were recorded between samples (Figure 8), with the control waffles showing a higher value than the waffles with added almond skin. This reduction in the b* coordinate, resulting from the addition of the skins, showed a reduction in the “yellow” colour of the control waffles to a darker shade.

**Figure 9:**
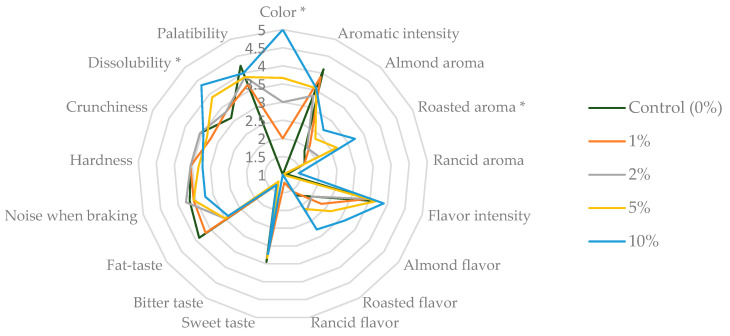
Sensory profile of the waffles after the elaboration of the quantitative descriptive analysis. The descriptors that presented significant differences (*p* ≤ 0.05, Duncan’s test) are marked with * (asterisk).

Finally, concerning brightness (L*), significant differences were recorded among all samples (Figure 8), with a reduction in this parameter as a result of the increase in the percentage of almond skin added to the waffle recipe; this decrease in brightness, as would be expected, decreased with the increasing skin content. Similarly, Pasqualone et al. [4] baked waffles with different amounts of almond skin added (0 g, 10 g, and 20 g for 100 g of cookie) and found that the colour of the waffles became progressively significantly darker as the level of skin addition increased. These data were proven sensorially, as the waffles were evaluated with “more colour” as the concentration of almond skin in their formulation increased (Figure 9 in the next discussion point).

### 2.5. Waffle Sensory Profile

Analysing the graph in Figure 9, we can see that the sensory profile of the waffles (the line connecting all the midpoints of the descriptors of the samples) was modified according to the percentage of almond skin added to their formulation.

After ANOVA (Duncan’s test for *p* ≤ 0.05), we found that the samples were statistically different in the descriptors “colour”, “roasted aroma”, and “dissolubility” (Figure 9). Regarding the “colour”, the tasters indicated that the waffle with 10% of the almond skin added was the most intense, and the least intense was the control waffle. This result was expected, since the almond skin has a brown colour, and is corroborated by the results of the instrumental colour evaluation.

Concerning the descriptor “roasted flavour”, the tasters noticed significant differences among the samples, with a lower “roasted flavour” for the control samples and the waffle with 1% almond skin, and a higher one for the waffle incorporated with 10% almond skin. The incorporation of almond skin increased this roasted flavour, even without an actual roasting of the samples, and is linked to the sensory properties of almond skin.

Regarding the “dissolubility”, we found that the waffle with 10% almond skin was significantly more dissoluble. We also verified that, like in the colour parameter, the dissolubility was directly proportional to the concentration of added almond skins. The addition of almond skin changed the rheological characteristics of the mixture used to make the waffles.

Finally, regarding the remaining descriptors, there were no significant differences. However, regarding the descriptor “roasted flavour”, there was a higher value for the sample with 10%. In the case of “fat-taste”, there was a higher value for the control sample and a lower one for the 10% almond skin, which indicates that the addition of almond skin may mask the fatty mouthfeel, since all formulations used the same amount of butter.

**Figure 10:**
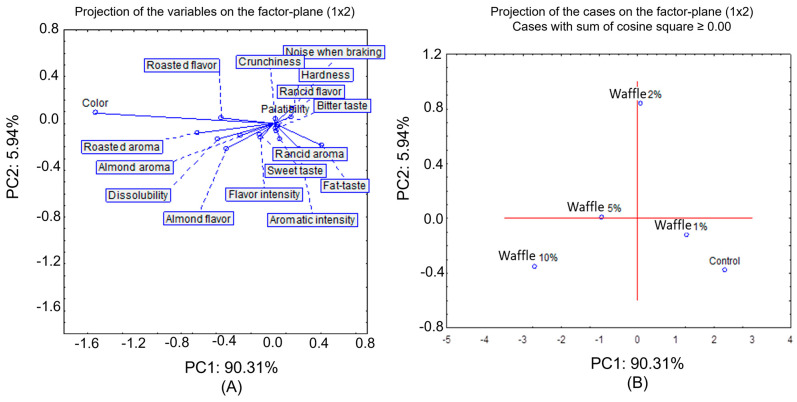
Principal component analysis (PCA) after quantitative descriptive analysis (QDA) of the waffle samples. (**A**) Representation of the descriptors in the two principal components PC1 and PC2. (**B**) Representation of the samples in the two principal components PC1 and PC2.

To better understand the relationship between the sensory descriptors and the sensorially evaluated waffle samples, a principal component analysis was performed (Figure 10). By analysing Figure 10, we can see that the samples were distributed over the four quadrants of the PCA. According to Table 2, we can see that the first component (Factor 1) had the most weight in the distribution of the samples. Thus, in the first and second quadrants were the 1%, 2%, and control samples, essentially characterised by the fat-taste descriptor (loading value of 0.41027, Table 2). In the third and fourth quadrants, the 5% and 10% samples were located. These samples were characterised by the descriptors colour, roasted aroma, almond and roasted flavour, and dissolubility. Overall, we can say that the preference of the tasters was for the 10% waffles.

### 2.6. Chemical and Sensory Parameters Correlation by PCA Analysis

**Figure 11:**
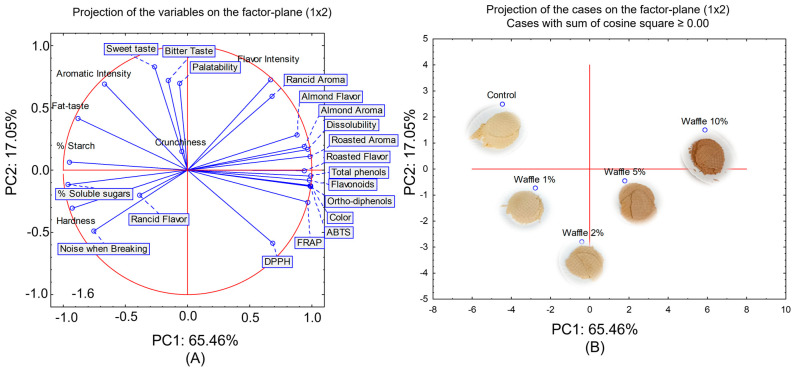
Principal component analysis (corr—PCA) considering all of the parameters evaluated. (**A**) Representation of the parameter (sensory and chemical) in the two principal components PC1 and PC2. (**B**) Representation of the samples in the two principal components PC1 and PC2.

Considering all the parameters evaluated, a new PCA was performed based on the correlations. The correlation matrix was singular, and all of the computations were based on generalised inverse. Figure 11A shows the representation of the sensory and chemical parameters assessed, while Figure 11B shows the representation of the samples in the two main components (PC1 and PC2), which accounted for more than 82% of the total variability explained in this analysis.

By analysing Figure 11, we can see that Factor 1 accounted for more than 65% of the total variability, while Factor 2 for more than 17%. The samples were distributed as they were when only the sensory descriptors were used to construct the PCA graphic representation. The 10% almond skin sample largely presented all nutraceutical properties, together with pleasant sensory descriptors such as almond aroma and flavour, roasted aroma and flavour, and dissolubility.

## 3. Materials and Methods

### 3.1. Almond Skin Extraction

The almond hull was removed, and the shell was separated from the kernel using a nutcracker. Subsequently, the kernels were blanched in boiling water at 95 °C for 3 min, and later, the skin was removed manually. Finally, the skins were oven-dried at 95–98 °C for 10 h until reaching a constant weight. The dried samples were packaged in vacuum bags and kept at room temperature until the extraction procedure.

### 3.2. Waffle Preparation

Waffles were prepared with 120 g of sugar, 4 eggs, 170 g of butter (melted and cooled), 1 tsp of baking powder, and 200 mL of water. All ingredients were carefully measured and mixed in a dish with a hand domestic mixer (Braun, GmbH, Kronberg Taunus, Germany). The previous mixture was divided into five parts and flour was added, namely, 50 g for the control samples and substituted by almond skin at 1%, 2%, 5%, and 10% for the remaining samples (0.5 g, 1 g, 2.5 g, and 5 g, respectively). Dough portions were applied to the centre of a Clatronic HA 3494 waffle maker (Kempen, Germany) and baked for approximately 1.5 min at 180 °C. After cooling, the waffles were hermetically packed and used in further analysis.

### 3.3. Preparation of Extracts for Bioactive Compounds and Antioxidant Assays

Extracts were prepared by weighing 40 mg of finely ground samples and thoroughly vortex-mixed with 1 mL of 70% methanol. These mixtures were heated at 70 °C for 30 min, centrifuged at 5000 rpm, and 4 °C for 15 min (Eppendorf Centrifuge 5804 R, Eppendorf AG, Hamburg, Germany), with the supernatants collected and filtered with Spartan filters (0.2 mm) to HPLC amber vials [31].

### 3.4. Bioactive Compounds: Total Phenolic Content, Total Flavonoid Content, and Ortho-Diphenols

The method for the quantification of the total phenolic content was adapted from Singleton et al. [32]. A total of 20 µL of the extract was mixed with 100 µL of Folin–Ciocalteu phenol reagent (1:10 in bidistilled H_2_O) and 80 µL of 7.5% Na_2_CO_3_ in a 96-well microplate (Multiskan^TM^ FC Microplate Photometer, Waltham, MA, USA). After incubation for 15 min at 45 °C in the dark, absorbance values against a blank were recorded at 765 nm in a microplate reader (BMG LABTECH, SPECTROstar Nano). A standard curve with gallic acid at different concentrations was performed and the total phenolic content results were expressed as mg gallic acid equivalent (GAE)/g of fresh weight (FW) as the mean ± standard deviation (SD) of three replicates.

The total flavonoid content was determined by spectrophotometry using the method of Dewanto et al. [33]. After the extraction procedure, 25 µL of the sample was taken and 100 µL of ultrapure water and 10 µL of 5% sodium nitrate (NaNO_3_) were added and mixed. The solution was in the dark at room temperature for 5 min. Next, 15 µL of 10% aluminium chloride (AlCl_3_) was added and mixed. The solution was allowed to stand in the dark at room temperature for 6 min. Furthermore, 50 µL of 1 M sodium hydroxide (NaOH), and 50 µL of ultrapure water were added and mixed. Finally, a calibration curve was performed, with a standard solution of catechin at different concentrations. The absorbance reading was performed in a microplate reader at a wavelength of 510 nm. The total flavonoid content was expressed as mg catechin equivalent (CE) g^−1^ fresh weight.

The method used for the determination of *ortho*-diphenols was adapted from Gutfinger [34] and Garcia et al. [35]. A total of 20 µL of the extract was mixed with 100 µL of ultra-pure water, 80 µL of phosphate buffer (pH 6.5, 0.1 M), and 160 µL of 5% sodium molybdate (Na_2_MoO_4_·2H_2_O) solution. After 15 min in the dark, the absorbance was measured at 370 nm against a blank reagent, with caffeic acid used as the standard to prepare a calibration curve and the *ortho*-diphenolic content expressed as caffeic acid equivalents per g of fresh weight (mg CAE/g FW) as the mean ± standard deviation (SD) of three replicates.

### 3.5. Antioxidant Activities (AA)

The method of Stratil et al. [36] was used to evaluate the 2,2-azino-bis (3-ethylbenzothiazoline 6-sulphonic) acid (ABTS) radical scavenging activity. An ABTS radical solution was prepared by mixing 7 mM of ABTS at pH 7.4 (5 mM NaH_2_PO_4_, 5 mM Na_2_HPO_4_, and 154 mM NaCl) with 2.5 mM K_2_S_2_O_8_. After overnight incubation in the dark at room temperature, this ABTS solution was diluted with ethanol until an absorbance of 0.70 ± 0.02 units at 734 nm was obtained. In each microplate well, 15 µL of the extract was mixed with 285 µL of freshly prepared ABTS solution, incubated at room temperature in the dark for 10 min, and the absorbance values were measured at 734 nm. The ABTS activity was expressed using the linear calibration curve of Trolox as g Trolox equivalent/g of fresh weight (FW).

The 2,2-diphenyl-1-picrylhydrazyl (DPPH) antioxidant activity assay was adapted from Bondet et al. [37], Sánchez-Moreno et al. [38], and Siddhraju et al. [39]. Briefly, 15 µL of the extract and 285 µL of freshly prepared methanolic radical DPPH solution (6 × 10^−5^ mol/L) were mixed in a 96-well microplate. The microplates were left for 30 min at room temperature and in the dark. The reduction in absorbance was measured at 517 nm and the DPPH activity was expressed using the linear calibration curve of Trolox as g Trolox equivalent/g FW.

The ferric-reducing antioxidant power (FRAP) assay was performed as described by Stratil et al. [36]. Briefly, a volume of 10 mM solution of 2,4,6-Tri (2-pyridyl)-S-triazine (TPTZ) in 40 mM HCl was mixed with the same volume of 20 mM FeCl_3_·6H_2_O and 10 times the volume of acetate buffer pH 3.6 (3.1 g sodium acetate and 16 mL acetic acid per L). A total of 275 µL of the Fe^3+^-TPTZ mixture was added with 25 µL of the extract and incubated. After 5 min, the absorbances were recorded at 593 nm, and FRAP was expressed from the linear calibration curve of Trolox as µg Trolox equivalent/g FW.

### 3.6. Extraction and Quantification of Soluble Sugars

Soluble sugars were determined by the method described by Irigoyen et al. [40]. Approximately 25 mg of the sample was placed in 5 mL of 80% ethanol (ethanol:water) at 80 °C. The reaction occurred for 1 h. Next, quantification was carried out, which was based on the colorimetric method with anthrone. A total of 100 µL of alcoholic extract and 1.5 mL of anthrone were added to a test tube. Afterwards, the tubes were vortexed, boiled for 10 min at 100 °C, and cooled on ice. A calibration curve was performed with a glucose solution; absorbance was read at a wavelength of 625 nm. In the end, the absorbance value was converted to µg/mL using a standard curve (0.05% glucose stock solution), and the sugar values were expressed as a percentage.

### 3.7. Extraction and Quantification of Starch

Starch was determined by the method described by Osaki et al. [41]. The supernatant of the sugars was eliminated, and the material was kept in the same test tube and 5 mL of 30% perchloric acid (perchloric acid: H_2_O) was added. The tubes were placed in a 60 °C water bath for 1 h. Next, we proceeded to quantification, where 150 µL of the extract and 1.5 mL of anthrone were added to each test tube. Subsequently, the tubes were vortexed, boiled for 10 min at 100 °C, and then cooled on ice. A calibration curve was performed with a glucose stock solution; absorbance was read at a wavelength of 625 nm. The absorbance value was converted to µg/mL by using a standard curve (0.05% glucose solution) and the starch values were expressed as a percentage.

### 3.8. Texture Evaluation

For texture evaluation, namely, the force required to break the waffle, a TA-XT plus Stable Micro Systems, UK texturometer with a 5 kg load cell was used. Tests were performed using a 2-mm diameter cylindrical stainless-steel probe. The test speed and distance travelled by the probe were 1 mm/s and 3 mm, respectively. The measurements were performed at room temperature and under constant light [42].

### 3.9. Colour Determination

The colour evaluation of the waffles was carried out on three waffles, with a reading on each face, using a Minolta CR 400 colorimeter. The colour was evaluated in a three-dimensional coordinate system using the L*, a*, b* model, in which colour is measured in three dimensions: luminosity (L*) and two chromatic coordinates (a* ranging from green to red and b* ranging from blue to yellow) [24].

### 3.10. Sensory Evaluation of Waffles by Quantitative Descriptive Analysis (QDA)

The sensory analysis was performed by a panel of nine tasters (seven women and two men) from UTAD, who are specialised in this type of evaluation. The samples were stored in adequate light and temperature conditions. The waffles were presented to the tasters on tasting plates (white Pyrex dishes), properly identified with random, alphanumeric codes, and evaluated on a scale of 1 to 5 points, where 1 is the descriptor is not present and 5 is its presence is the maximum. Mineral water was provided for the tasters to clean their palates between samples. The tasting sheet was adapted from Civille et al. [43] and Ormenese et al. [44], and the descriptors chosen were appearance (colour), aroma (intensity, almond, roasted, mustiness/rancid), flavour (intensity, almond, roasted, mustiness, sweet, bitter, fat-taste), and hand/oral texture (noise when broken, hardness, crunchiness, dissolubility, palatability).

### 3.11. Data Analysis

All of the experiments were performed at least in triplicate to ensure statistical representation. The results were submitted to analysis of variance (ANOVA), Duncan’s tests (sensory analysis data), and Tukey’s tests (physicochemical analyses) at 5% significance. The evaluation of the sensory profile of the samples was carried out through the average of the descriptors, represented in a spider graph constructed in Excel (Microsoft software). Principal component analysis (PCA), based on covariance matrices (sensory data only) or correlation matrices (all data), was also performed. All analyses were conducted using the Statistica 2020 software (StatSoft Inc., Tulsa, OK, USA, 2020).

## 4. Conclusions

The aim of this work was the evaluation of the effect of almond skin incorporation in waffles. The results showed that the ingredient studied is rich in bioactive compounds and can be used in the preparation of waffles or other food formulations, since it can improve their organoleptic and chemical characteristics. The waffle preparation that showed higher levels of phenolic compounds as well as higher antioxidant activity was the waffle with higher almond skin supplementation.

Thus, the study shows that almond skins can be used in the formulation of new, healthier food products, particularly concerning the content of soluble sugars, starch, phenolic compounds, and antioxidant capacity, but that they are also able to improve their sensory characteristics. It can be used as a functional ingredient, improving the nutritional and health-promoting properties and organoleptic characteristics of waffles. More detailed studies regarding in vitro and in vivo digestibility assays, potential biological effects as well as the techno-functional properties should be performed.

## Figures and Tables

**Table 1:** Weight (mean ± SD) of the waffles with different percentages of almond skin.

Samples	Mean Weight ± Standard Deviation
Control	10.25 ± 1.99 g
1%	8.34 ± 0.81 g
2%	7.73 ± 0.80 g
5%	8.78 ± 0.29 g
10%	7.16 ± 1.40 g

**Table 2:** Loadings of the sensory attributes in the two principal components (Factor 1 and Factor 2). In bold are the loadings greater than 0.4.

Descriptors	Factor 1	Factor 2
**Colour**	**−1.53094**	0.088359
Aromatic Intensity	0.21600	−0.219561
Almond Aroma	0.28705	−0.102452
**Roasted Aroma**	**−0.65894**	−0.082851
Rancid Aroma	−0.12207	−0.99538
Flavour Intensity	−0.11662	−0.119623
**Almond Flavour**	**−0.41306**	−0.214428
**Roasted Flavour**	**−0.45348**	0.047448
Rancid Flavour	0.03684	−0.013759
Sweet taste	0.04925	−0.135587
Bitter Taste	0.01238	−0.047488
**Fat Taste**	**0.41027**	−0.189357
Noise when Breaking	0.15110	0.132059
Hardness	0.14321	0.058049
Crunchiness	0.01060	0.039401
**Dissolubility**	**−0.48132**	−0.132656
Palatability	0.02239	−0.068781

## Data Availability

All of the data are available in the article.

## Sample Availability

Not available.

## References

[1] Esfahlan J., Jamei R. (2010). The importance of almond (*Prunus amygdalus* L.) and its by-products. Food Chem..

[2] Prgomet I., Goncalves B., Domínguez-Perles R., Pascual-Seva N., Barros A. (2017). Valorization challenges to almond residues: Phytochemical composition and functional application. Molecules.

[3] Garrido I., Monagas M., Gómez-Cordovés C., Bartolomé B. (2008). Polyphenols and antioxidant properties of almond skins: Influence of industrial processing. J. Food Sci..

[4] Pasqualone A., Laddomada B., Boukid F., Angelis D., Summo C. (2020). Use of almond skins to improve nutritional and functional properties of biscuits: An example of upcycling. Foods.

[5] Valdés A., Vidal L., Beltrán A., Canals A., Garrigós M.C. (2015). Microwave-assisted extraction of phenolic compounds from almond skin byproducts (*Prunus amygdalus*): A multivariate analysis approach. J. Agric. Food Chem..

[6] Malayil S., Surendran N., Kate K., Satyavolu J. (2022). Impact of acid hydrolysis on composition, morphology and xylose recovery from almond biomass (skin and shell). Bioresour. Technol. Rep..

[7] Loizzo R., Tundis R., Leporini M., D’Urso G., Gagliano-Candela T., Sottile F. (2021). Almond (*Prunus dulcis* cv. Casteltermini) skin confectionery by-products: A new opportunity for the development of a functional blackberry (*Rubus ulmifolius* Schott) Jam. Antioxidants.

[8] Arena A., Bisignano C., Stassi G., Filocamo A., Mandalari G. (2015). Almond skin inhibits HSV-2 replication in peripheral blood mononuclear cells by modulating the cytokine network. Molecules.

[9] Mandalari G., Tomaino A., Arcoraci T., Martorana M., Rich T., Bisignano C., Saija A., Dugo P., Cross L., Waldron W. (2010). Characterization of polyphenols, lipids, and dietary fibre from almond skins (*Amygdalus communis* L.). J. Food Compos. Anal..

[10] Reyes-De-Corcuera J., Cavalieri P., Powers R., Reyes I., Corcuera D., Heldman D.R., Moraru C.I. (2010). Chapter 5—Blanching. Encyclopedia of Agricultural, Food, and Biological Engineering.

[11] Mandalari G., Tomaino A., Rich T., lo Curto R., Arcoraci T., Martorana M., Bisignano C., Saija A., Parker L., Waldron W. (2010). Polyphenol and nutrient release from the skin of almonds during simulated human digestion. Food Chem..

[12] Arena A., Bisignano C., Stassi G., Mandalari G., Wickham S., Bisignano G. (2010). Immunomodulatory and antiviral activity of almond skins. Immunol. Lett..

[13] Hamdani M., Wani A., Bhat A. (2021). Pasting, rheology, antioxidant and texture profile of gluten-free cookies with added seed gum hydrocolloids. Food Sci. Technol. Int..

[14] Lusk L. (2019). Consumer beliefs about healthy foods and diets. PLoS ONE.

[15] Galanakis C.M. (2021). Functionality of Food Components and Emerging Technologies. Foods.

[16] Mahloko M., Silungwe H., Mashau E., Kgatla E. (2019). Bioactive compounds, antioxidant activity and physical characteristics of wheat-prickly pear and banana biscuits. Heliyon.

[17] Olagunju A., Omoba O., Enujiugha V., Aluko R. (2018). Development of value-added nutritious crackers with high antidiabetic properties from blends of Acha (*Digitaria exilis*) and blanched Pigeon pea (*Cajanus cajan*). Food Sci. Nutr..

[18] Ismail T., Akhtar S., Riaz M., Ismail A. (2014). Effect of pomegranate peel supplementation on nutritional, organoleptic and stability properties of cookies. Int. J. Food Sci. Nutr..

[19] Dhillon J., Newman J., Fiehn O., Ortiz R. (2023). Almond consumption for 8 weeks altered host and microbial metabolism in comparison to a control snack in young adults. J. Am. Nutr. Assoc..

[20] Szymanowska U., Karaś M., Złotek U., Jakubczyk A. (2021). Effect of fortification with raspberry juice on the antioxidant and potentially anti-inflammatory activity of waffles subjected to in vitro digestion. Foods.

[21] Szymanowska U., Karaś M., Bochnak-Niedźwiecka J. (2021). Antioxidant and anti-inflammatory potential and consumer acceptance of waffles enriched with Freeze-Dried Raspberry Pomace. Appl. Sci..

[22] Urganci U., Fatma K. (2021). Quality characteristics of biscuits fortified with pomegranate peel. Akad. Gıda.

[23] Salem R., El-Sahy M., Sulieman M., Gouda R. (2020). Use of tomato pomace, mango seeds kernel and pomegranate peels powders for the production of functional biscuits. J. Agric. Res..

[24] Oliveira I., Meyer A., Afonso S., Ribeiro C., Gonçalves B. (2017). Morphological, mechanical and antioxidant properties of Portuguese almond cultivars. J. Food Sci. Technol..

[25] Oliveira I., Meyer S., Afonso S., Sequeira A., Vilela A., Goufo P., Trindade H., Gonçalves B. (2020). Effects of different processing treatments on almond (*Prunus dulcis*) bioactive compounds, antioxidant activities, fatty acids, and sensorial characteristics. Plants.

[26] Ogunjobi K., Ogunwolu O. (2010). Physicochemical and sensory properties of cassava flour biscuits supplemented with cashew apple powder. J. Food Technol..

[27] Hooda S., Jood S. (2005). Organoleptic and nutritional evaluation of wheat biscuits supplemented with untreated and treated fenugreek flour. Food Chem..

[28] Ng H., Robert D., Ahmad W., Ishak W. (2017). Incorporation of dietary fiber-rich oyster mushroom (*Pleurotus sajor-caju*) powder improves postprandial glycaemic response by interfering with starch granule structure and starch digestibility of biscuit. Food Chem..

[29] Sudha L., Vetrimani R., Leelavathi K. (2007). Influence of fibre from different cereals on the rheological characteristics of wheat flour dough and on biscuit quality. Food Chem..

[30] Ajila M., Leelavathi S., Rao P. (2008). Improvement of dietary fiber content and antioxidant properties in soft dough biscuits with the incorporation of mango peel powder. J. Cereal Sci..

[31] Oliveira I., Meyer A., Afonso S., Aires A., Goufo P., Trindade H., Gonçalves B. (2019). Phenolic and fatty acid profiles, *α*-tocopherol and sucrose contents, and antioxidant capacities of understudied Portuguese almond cultivars. J. Food Biochem..

[32] Singleton L., Rossi A. (1965). Colorimetry of total phenolics with phosphomolybdic-phosphotungstic acid reagents. Am. J. Enol. Vitic..

[33] Dewanto V., Wu X., Adom K., Liu H. (2002). Thermal processing enhances the nutritional value of tomatoes by increasing total antioxidant activity. J. Agric. Food Chem..

[34] Gutfinger T. (1981). Polyphenols in olive oils. J. Am. Oil Chem. Soc..

[35] Garcia B., Coelho J., Costa M., Pinto J., Paiva-Martins F. (2013). A simple method for the determination of bioactive antioxidants in virgin olive oils. J. Sci. Food Agric..

[36] Stratil P., Klejdus B., Kubáň V. (2006). Determination of total content of phenolic compounds and their antioxidant activity in vegetables evaluation of spectrophotometric methods. J. Sci. Food Agric..

[37] Bondet V., Brand-Williams W., Berset T. (1995). Kinetics and mechanisms of antioxidant activity using the DPPH. Free radical method. Food Sci. Technol..

[38] Sánchez-Moreno C., Larrauri A., Saura-Calixto F. (1998). A procedure to measure the antiradical efficiency of polyphenols. J. Sci. Food Agric..

[39] Siddhraju P., Becker K. (2003). Antioxidant properties of various solvents extracts of total phenolic constituents from three different agroclimatic origins of drumstick tree (*Moringa oleifera* Lam.) leaves. J. Agric. Food Chem..

[40] Irigoyen J., Einerich W., Sánchez-Díaz M. (1992). Water stress induced changes in concentrations of proline and total soluble sugars in nodulated alfalfa (*Medicago sativa*) plants. Physiol. Plant..

[41] Osaki K., Doi M. (1991). On the concentration gradient of polymer solution in a rotating rheometer. J. Rheol..

[42] Fragoso P. (2016). Desenvolvimento de Bolachas com Incorporação de Diferentes Microalgas. Ph.D. Thesis.

[43] Civille V., Lapsley K., Huang G., Yada S., Seltsam J. (2010). Development of an almond lexicon to assess the sensory properties of almond varieties. J. Sens. Stud..

[44] Ormenese C., Abreu N., Coelho D., Silva D. (2001). Perfil sensorial e teste de consumidor de biscoito recheado sabor chocolate. Bol. Cent. Pesqui. Process. Aliment..

